# The TRAIN Health Awareness Clinical Trial: Baseline Findings and Cardiovascular Risk Management in Aortic Dissection Patients

**DOI:** 10.1055/a-2524-4772

**Published:** 2025-02-17

**Authors:** Nora Bacour, Simran Grewal, Aytug U. Tirpan, Rutger Theijse, Olivia Van Erp, Robert J.M. Klautz, Natzi Sakalihasan, Rebecka Hultgren, Nimrat Grewal

**Affiliations:** 1 Department of Cardiothoracic Surgery, Amsterdam University Medical Center, Amsterdam, The Netherlands; 2 Department of Orthopaedic Surgery, Amsterdam University Medical Center, Amsterdam, The Netherlands; 3 Department of Cardiothoracic Surgery, Leiden University Medical Center, Leiden, The Netherlands; 4 Department of Cardiovascular and Thoracic Surgery, CHU Liège, University of Liège, Liège, Belgium.; 5 Department of Vascular Surgery, Karolinska University Hospital, Stockholm, Sweden; 6 Department of Molecular Medicine and Surgery, Karolinska Institutet, Stockholm, Sweden

**Keywords:** Type A aortic dissection, cardiovascular risk management, eHealth Interventions, lifestyle modifications, postoperative outcomes

## Abstract

**Background/Objective:**

Acute Type A aortic dissection (ATAAD) is a life-threatening condition requiring timely surgical intervention. Despite successful surgery, postoperative outcomes are frequently suboptimal due to the high frequency of cardiovascular risk factors. This study examines baseline cardiovascular risk factors in a population of ATAAD patients in the Netherlands. Additionally, this study outlines the protocol for a randomized controlled trial, designed to improve postoperative management.

**Methods:**

Baseline data were collected from patients with ATAAD. Data were gained through the Stichting Aorta Dissectie Nederland, a Dutch association for aortic dissection patients. The data included information on cardiovascular risk factors and health-related quality of life. A survey was further conducted, to gain more insights into the ATAAD postoperative care experiences of cardiac and vascular surgeons.

**Results:**

Among the 50 ATAAD patients in our study, we found significant cardiovascular risk factors, including smoking (36.7%), obesity (34.2%), and hypertension (51.3%). In the surgeon survey ( *N* = 48), 84% of respondents highlighted the significance of lifestyle changes for patients, underscoring the need for individualized risk management. These findings underscore the need for tailored postoperative management programs aimed at improving patient outcomes.

**Conclusion:**

The results of our study highlight that ATAAD patients require comprehensive postoperative care management strategies. The ultimate goal is to enhance long-term patient outcomes and improve health-related quality of life. To address this need, the TRAIN (Targeted caRdiovAscular rIsk reductioN) Health Awareness platform seeks to implement personalized eHealth-based lifestyle interventions.

## Introduction

Acute Type A aortic dissection (ATAAD) is a potentially fatal condition, requiring prompt surgical intervention. 1 While successful aortic surgery can stabilize patients, many ATAAD survivors experience considerable challenges in the postoperative period due to a number of cardiovascular risk factors. 2 These factors, including obesity, hypertension, physical inactivity, and dyslipidemia can significantly affect long-term results, necessitating comprehensive postoperative management strategy aimed at improving survival and quality of life.

Current postoperative care tends to prioritize surgical repair and the immediate postsurgery period, but long-term management of cardiovascular risk factors is often neglected. 3 This gap in care can lead to suboptimal postoperative outcomes, such as recurrent cardiovascular events and reduced quality of life. 4 To address this, postoperative therapy for ATAAD patients should focus not only on monitoring the surgical site, but also reducing modifiable risk factors. Incorporating lifestyle interventions, such as weight management, exercise, and blood pressure control, a multidisciplinary care plan could help to mitigate the abovementioned risks.

As the prevalence of cardiovascular risk factors remains significant, along with its association with mortality, 5 the development of a comprehensive, multidisciplinary approach to postoperative care becomes imperative. Such an approach should integrate lifestyle modifications, personalized interventions, continuous monitoring, and guidance to address the specific needs of each patient. Utilizing eHealth technology presents a promising method to improve patient involvement and compliance with suggested lifestyle modifications. 6

To understand barriers of postoperative care for ATAAD patients, we carried out a survey among cardiac and vascular surgeons during the 8th International Meeting on Aortic Diseases. 7 The survey questioned the health care professionals about the importance of personalized risk management, lifestyle modifications, and continuous monitoring in the management of aortopathy. We further collected baseline data from ATAAD patients affiliated with Stichting Aorta Dissectie Nederland (SADN), a Dutch association for patients who have survived an aortic dissection. Information on patient demographics, perceived health, lifestyle factors, and other relevant health indicators were all collected. Such comprehensive baseline data from the SADN cohort is extremely significant as it allows us to identify critical targets for intervention and develop tailored strategies to improve patient outcomes.

In this paper, we describe the combined baseline findings from the SADN population and the insights from the surgeon survey, which were presented during the 8th Annual International Meeting on Aortic Diseases (IMAD) and which have been instrumental in providing a robust foundation for developing the TRAIN (Targeted caRdiovAscular rIsk reductioN) Health Awareness Clinical Trial. This multicenter prospective randomized controlled trial will be conducted at the Amsterdam University Medical Center and Leiden University Medical Center (LUMC). The trial aims to assess the impact of a customized, multidisciplinary health quality improvement program on cardiovascular risk factors, cardiovascular events, and health-related quality of life in ATAAD patients.

## Materials and Methods

### Study Design

This study consists of two primary components: the collection of baseline data from ATAAD patients affiliated with SADN and a survey conducted among cardiac and vascular surgeons at the 8th Annual IMAD. 7

### Ethical Considerations

This study was conducted as part of the biobank protocol with reference number: B21.051/MS/ms.

The Medical Ethical Committee of LUMC reviewed and approved the study protocol prior to the start of the inclusions. Furthermore, all participants were required to provide informed consent before participating in the study. Ethical principles outlined in the Declaration of Helsinki and Good Clinical Practice guidelines were upheld during the entirety of the study, ensuring the confidentiality and anonymity of all collected data.

### Stichting Aorta Dissectie Nederland Baseline Data Collection

#### Participants

Patients affiliated with SADN who had a history of ATAAD and were willing to participate were included in the study.

Inclusion criteria included:

- Age ≥ 18 years.
- Previous diagnosis of ATAAD.
- Enrollment in SADN.

Exclusion criteria included:

- Comorbid conditions that could interfere with the results of the study.
- Inability to provide informed consent.

### Survey Instrument

A comprehensive survey was created with the objective of collecting specific data on the participants' demographics, health status, and additional lifestyle factors. Questions in the following domains were included in the survey:

- Demographics: age, gender, education level, and employment status.
- Health status: medical history, including the prevalence of hypertension, diabetes, and cholesterol levels.
- Lifestyle factors: physical activity levels, smoking habits, dietary habits, and alcohol consumption.
- Health indicators: sleep quality, mental health status, mobility limitations, and self-care abilities.

### Data Collection Procedure

Participants were contacted through the SADN network and invited to participate in our study by completing a web-based survey. Paper copies of the survey were distributed among participants who indicated their preference for this format. All participants provided informed consent before participating in the survey. To optimize participation, reminders were given to nonrespondents over the 6-month data-collecting period.

### International Meeting on Aortic Diseases Surgeon Survey

Cardiac and vascular surgeons who attended the IMAD and consented to participate in the survey were included. There were no specific exclusion criteria for surgeons beyond their attendance at the conference and willingness to participate. When surgeons expressed their interest, they were given access to the survey through an online survey link.

The survey was designed with the objective of gathering insights from surgeons on the challenges and best practices in the postoperative management of ATAAD patients. Questions in the following domains were included in the survey:

- Experiences: common postoperative complications in post-ATAAD patients.
- Management strategies: surgeons views on critical factors in postoperative management.
- Barriers to effective management: challenges faced in implementing effective postoperative care strategies.

### Data Analysis

Descriptive statistics were used to describe the baseline characteristics of the SADN patients and responses from the surgeons' survey. The SADN survey was used to assess key variables in postdissection patients. The IMAD survey was used to thematically analyze recurring themes and insights on postoperative management strategies and challenges faced by cardiothoracic surgeons throughout the world. All data were analyzed using SPSS-27 software.

## Results

### Demographic Characteristics

The baseline survey was conducted among 50 patients with a history of ATAAD. These patients, whose ages ranged from 35 to 82 years with a mean age of 59 years, predominantly comprised males (75%) compared with females (25%).

### Health and Lifestyle Factors

The responses of our survey revealed that cardiovascular risk factors are quite common postdissection. First, hypertension was reported by 51.3% of the patients and the mean blood pressure of the participants was 140/90 mm Hg, which is considered a key risk factor for adverse cardiovascular outcomes. 8 9 Furthermore, 34.2% of respondents had a body mass index of 30 or above, indicating that they are obese. These results highlight the seriousness of obesity in this patient group, which is linked to numerous health issues, such as an elevated risk of cardiovascular disease. 10 11 Additionally, diabetes was reported by 12% of patients, adding to this cardiovascular risk burden. However, the treatment status of patients for these cardiovascular conditions—such as the use of antihypertensives, cholesterol-lowering medications, or smoking cessation protocols—was not consistently available in our dataset.

Physical inactivity was another prevalent issue, as 62% of respondents indicated they were not moderately active for more than 30 minutes a day. Both cardiovascular health and general well-being are greatly at risk from this sedentary lifestyle. 12 13 Sleep quality was another major issue, as 48% of patients reporting sleep disturbances and the average nightly sleep duration was 6 hours. These problems are well-known to negatively affect cardiovascular health and general quality of life. 14 15

In addition, 36.7% of patients reported being current or former smokers and 40% of patients had elevated cholesterol levels, both modifiable risk factors that further exacerbate their cardiovascular risk profile. 16 17 Dietary habits were another area of concern as many patients reported eating fewer fruits and vegetables than was advised. Addressing this through targeted interventions that include both pharmacological treatment and lifestyle changes, such as an improved diet and increased physical activity, is critical for managing cardiovascular risk and improving overall health outcomes. 17 18

#### Mental Health and Mobility

Mobility and mental health were also important issues. A notable proportion of patients reported experiencing anxiety and depression, highlighting the need for postoperative therapy to incorporate comprehensive mental health support. Additionally, mobility issues were reported by 25% of the patients, and 15% had difficulties with daily activities and self-care. These findings suggest that thorough rehabilitation programs are necessary to promote the patients' functional recovery.

**Fig. 1:**
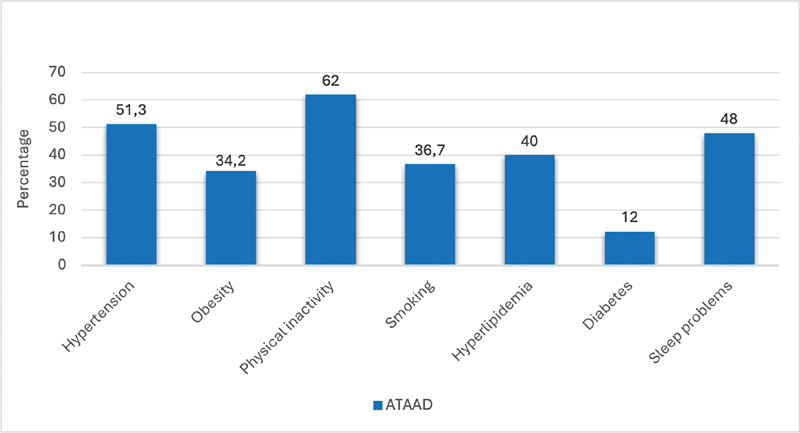
Baseline cardiovascular risk factors of ATAAD patients. This figure provides a detailed overview of the prevalence of various cardiovascular risk factors among ATAAD patients, including hypertension, obesity, physical inactivity, smoking, hyperlipidemia, diabetes, and sleep problems. The high prevalence of these modifiable risk factors highlights the urgent need for comprehensive risk management strategies to improve patient outcomes. ATAAD, acute Type A aortic dissection.

An overview of the risk factors is presented in Fig. 1 .

#### Insights from the International Meeting on Aortic Diseases Surgeon Survey

**Fig. 2:**
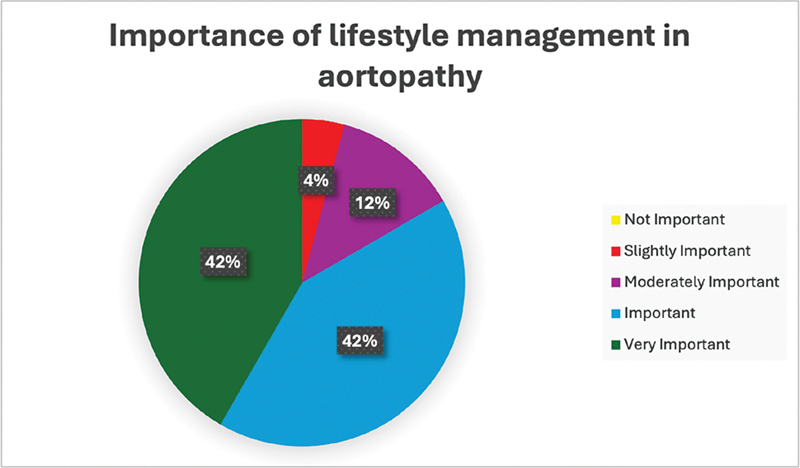
Importance of lifestyle interventions in the treatment of thoracic aortopathy according to surgeons. This figure illustrates the distribution of surgeons' opinions on the importance of various lifestyle interventions, such as dietary changes, physical activity, and smoking cessation, in managing thoracic aortopathy. The majority of surgeons rated these interventions as important or very important, underscoring their perceived value in improving patient outcomes.

In addition to the patient survey, insights from the IMAD were gathered through a survey of cardiovascular surgeons ( *N* = 48). These surgeons underlined the significance of individualized risk management programs that are catered to the unique characteristics of each patient. They highlighted the critical role of lifestyle modifications (>84% considers important), including dietary changes, increased physical activity, smoking cessation, and stress management, in the postoperative care of ATAAD patients ( Fig. 2 ).

**Fig. 3:**
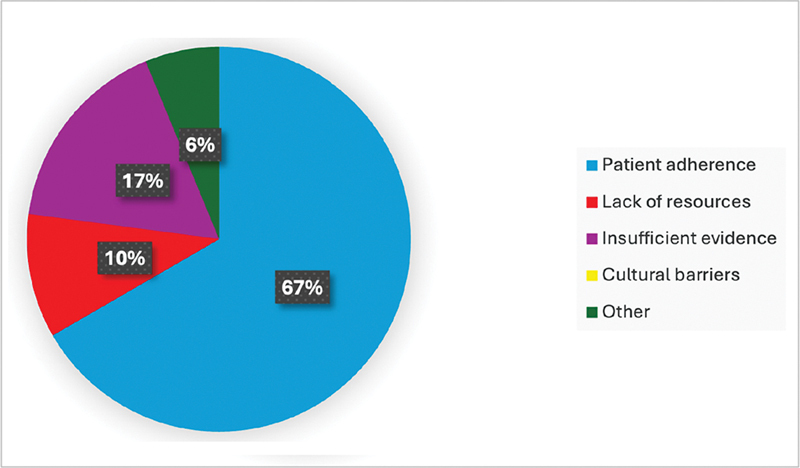
Barriers faced by surgeons in implementing lifestyle interventions in patients with thoracic aortopathy. This figure identifies the main challenges surgeons encounter when trying to implement lifestyle interventions for their patients. Common barriers include patient adherence, lack of resources, insufficient evidence, cultural barriers, and other unspecified issues. Understanding these barriers is crucial for developing effective strategies to support lifestyle modifications in clinical practice.

**Fig. 4:**
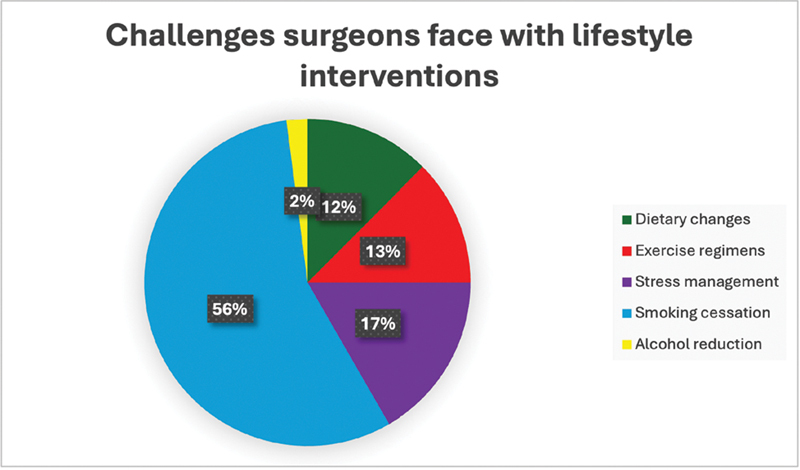
Specific lifestyle interventions that surgeons find most challenging to implement in patients with thoracic aortopathy. This figure highlights the specific lifestyle changes that surgeons find difficult to enforce, such as dietary changes, exercise regimens, stress management, smoking cessation, and alcohol reduction. Smoking cessation and stress management were identified as particularly challenging, indicating areas where additional support and resources may be needed.

The surgeons also emphasized the importance of continuous evaluation of cardiovascular risk factors in post-AATD patients to enhance patient outcomes. They promoted the adoption of digital health tools to help with routine follow-ups and early identification of potential complications. Nonetheless, they expressed their concerns about several barriers to successful postoperative treatment, such as patients' failure to follow recommended lifestyle modifications, a lack of resources for ongoing observation, and a lack of established standards for long-term care ( Fig. 3 ). Of all lifestyle interventions, smoking cessation and stress management were identified as the most challenging to implement ( Fig. 4 ). These challenges likely stem from the need for sustained behavioral changes, which require significant patient engagement and support. These combined findings from the SADN patient survey and the insights from the IMAD surgeon survey provide a comprehensive understanding of the current state of postoperative care for ATAAD patients. They underscore the need for targeted interventions to address the high prevalence of modifiable cardiovascular risk factors and the barriers to effective management ( Figs. 2 3 4 ).

## Discussion

The findings of our study indicate that individuals with ATAAD have substantial cardiovascular risk factors, necessitating all-encompassing care approaches ( Fig. 1 ). The high prevalence of hypertension (51.3%) is particularly notable, as it is a well-established risk factor for an aortic dissection. 8 19 The mean blood pressure of 140/90 mm Hg among respondents indicates suboptimal control of hypertension, necessitating more aggressive management strategies. Our findings also revealed that a substantial proportion of patients was obese (34.2%), which is associated with a variety of adverse cardiovascular outcomes, including increased blood pressure, dyslipidemia, and insulin resistance. 10

It is alarming that 62% of respondents reported poor levels of physical exercise because physical inactivity is a modifiable risk factor for cardiovascular and aortic illnesses. 12 20 21 In response to these poor levels of physical exercise, regular exercise should be encouraged to reduce the overall cardiovascular risk in post-AATD patients. Additionally, as smoking is linked to vascular inflammation, reduced aortic elasticity, and accelerated atherosclerosis, these patients should receive guidance accordingly to reduce this strain on their individual risk levels. 22 23 24

Elevated cholesterol levels remained common within our study population, consistent with the literature that identifies hyperlipidemia as a key contributor to atherosclerosis and the resulting aortic complications. 25 26 Twelve percent of respondents reported having diabetes. This introduces additional complexity, as diabetes is linked to accelerated atherosclerosis and increased vascular stiffness. 27

Previous research indicates that suboptimal sleep has also been linked to suboptimal recovery following surgical intervention. 28 In terms of cardiovascular disease, associations have been identified with underlying conditions such as sleep apnea. 29

As our study cohort revealed that sleep disturbances were reported by 48% of patients, future research into sleep quality in AATD patients and effective treatments could greatly enhance sleep. Addressing sleep quality could therefore be an integral part of a holistic approach to cardiovascular risk management, thereby improving overall recovery. Mobility issues and difficulties with daily activities reported by 25 and 15% of patients, respectively, highlight the impact of aortic disease on quality of life and functional status, emphasizing the need for comprehensive rehabilitation programs.

### Comparison with Literature

The findings from the SADN survey are consistent with existing literature on cardiovascular risk factors in aortic dissection patients. 30 Hypertension, as identified in this study, is frequently reported as a predominant risk factor in similar cohorts. 30 A study by Rueda-Ochoa et al 31 found comparable hypertension rates, underscoring the necessity of effective blood pressure management in preventing aortic events. 8

The high prevalence of smoking in our cohort is also supported by previous studies. Dolmaci et al 32 identified smoking as a crucial risk factor for aortic aneurysms and dissections, which aligns with our findings. The significant presence of hyperlipidemia among respondents' parallels studies that link elevated cholesterol levels to increased aortic stiffness and susceptibility to dissections. 25 33

### Insights from Cardiovascular Surgeons

Insights gained from cardiovascular surgeons at the IMAD conference highlight the importance of developing tailored risk management strategies. The surgeons that participated in our study underlined the need of incorporating lifestyle modifications into standard care protocols. This could entail developing personalized guidelines for dietary changes and increased physical activity. This holistic approach is crucial for addressing the multifaceted nature of cardiovascular risk factors in thoracic aortopathy patients. The necessity of continuous monitoring of cardiovascular risk factors was a key point highlighted by the surgeons. Risk factors can be identified early and handled timely by routinely checking in with patients and tracking individual health indicators. In turn, this will help improve patient outcomes. The integration of eHealth tools, such as the TRAIN mobile application used in the TRAIN Health Awareness Clinical Trial, offers a promising solution for continuous patient engagement and monitoring.

### Implications for Clinical Practice

The baseline findings from the SADN survey highlight the high prevalence of modifiable risk factors such as hypertension, obesity, smoking, and physical inactivity. These findings highlight urgent need for a comprehensive, multidisciplinary approach to managing cardiovascular risk factors in ATAAD patients. Components of the management strategy could entail lifestyle modifications, regular monitoring, and patient education.

**Fig. 5:**
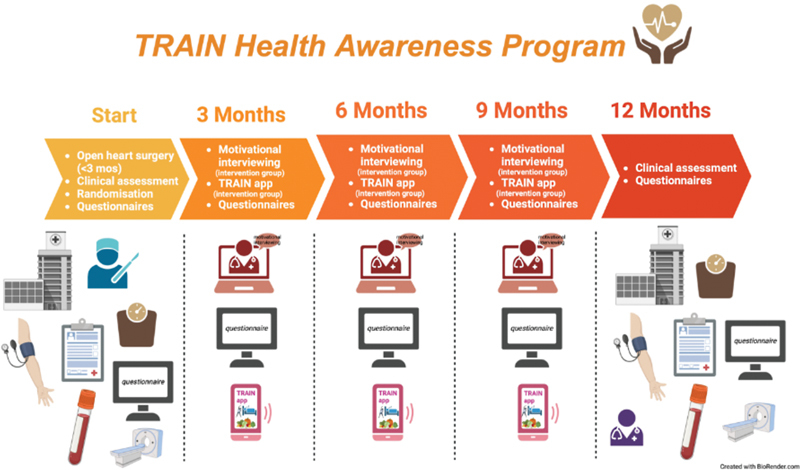
Protocol of the Health Awareness Clinical Trial. This figure outlines the structure, key components, and timeline of the TRAIN Health Awareness Clinical Trial. The trial includes: (1) patient engagement strategies: utilizing eHealth technologies for real-time tracking and educational materials. (2) Continuous monitoring: regular follow-ups and remote monitoring of cardiovascular risk factors. (3) Personalized interventions: tailored lifestyle modification plans including diet, exercise, and smoking cessation. (4) Multidisciplinary approach: collaboration among various healthcare professionals to provide comprehensive care. (5) Health quality improvement program: structured program aimed at reducing risk factors and enhancing quality of life. The timeline spans from initial patient enrollment and baseline assessments, through continuous monitoring and intervention phases, to follow-up evaluations at 6 weeks, 3 months, and 6 months of postintervention. The goal of the trial is to improve postoperative outcomes and quality of life for ATAAD patients through a holistic, patient-centered approach. ATAAD, acute Type A aortic dissection.

Our findings informed the development of the TRAIN Health Awareness Clinical Trial, a proposed multicenter randomized controlled trial aimed at assessing the impact of a customized, multidisciplinary health quality improvement program on cardiovascular risk factors, cardiovascular events, and health-related quality of life in ATAAD patients (protocol presented in Fig. 5 ). Using eHealth interventions can improve patient engagement and adherence to lifestyle changes by offering personalized feedback and tracking individual progress. This could ultimately result in more effective management of cardiovascular risk factors.

### Multidisciplinary Aortic Centers

The complexity of managing thoracic aortic illness highlights the necessity for interdisciplinary aortic centers. Such centers can integrate genetic counseling and testing, particularly for patients with connective tissue illnesses and those who have a family history of dissections. Genetic testing can provide crucial insights for patient monitoring and follow-up, helping tailor individual management plans.

Additionally, lifestyle recommendations ought to be a standard component of patient care, with an emphasis on tailored therapies that target particular risk factors and promote overall cardiovascular health. These centers can serve as hubs for comprehensive care, combining clinical expertise with ongoing research to improve patient outcomes.

### Genetic Testing and Lifestyle Recommendations

Another recommendation would be to adopt routine genetic testing for patients with a family history of dissection and young patients with thoracic aortic disease to identify those at high risk. By using this method, negative occurrences may be avoided by early interventions and customized monitoring plans. Additionally, personalized lifestyle recommendations based on genetic and clinical data can further enhance patient care.

### Future Directions

Future research should focus on longitudinal studies to evaluate the long-term impact of lifestyle interventions on patient outcomes as proposed in Fig. 5 . Further, investigating the genetic predispositions and pathophysiological mechanisms underlying thoracic aortopathy could offer insights into developing targeted therapies and preventive measures, exploring the role of novel biomarkers in risk stratification and monitoring could further refine personalized management strategies.

### Limitations

It is critical to recognize the limitations of our research. As our study relies on self-reported data from our participants, some bias and inaccuracies may be introduced. The survey's cross-sectional design restricts the capacity to draw conclusions about causality. Furthermore, the study population is limited to patients associated with the SADN, which may not be representative of the broader ATAAD patient population.

## Conclusions

The findings of our study underscore the importance of comprehensive cardiovascular risk management in post-ATAAD patients. The baseline results show that smoking, obesity, physical inactivity, and hypertension are important risk factors that require focused interventions. According to cardiovascular surgeons, individualized risk management plans that incorporate lifestyle changes and ongoing observation are necessary. The TRAIN Health Awareness Clinical Trial is an excellent chance to fill in the gaps in the existing management of ATAAD. Future research should focus on evaluating the long-term impact of these interventions and refining strategies to integrate them into standard care.
